# The complete chloroplast genome of *Artocarpus tonkinensis*, a tree native to China with diverse beneficial medicinal applications

**DOI:** 10.1080/23802359.2021.1997116

**Published:** 2021-12-09

**Authors:** Li-Ming Tang, Xiu Liu, Jian-Yong Lin, Bo Qin, Chong-Zheng Chen

**Affiliations:** a Forestry Department of Guangxi, Nanning, China; b Guangxi Forestry Research Institute, Nanning, China

**Keywords:** Chloroplast genome, *Artocarpus tonkinensis*, phylogenetic analysis

## Abstract

In the present study, we announce the first complete chloroplast genome sequence of *Artocarpus tonkinensis*, a tree native to China with diverse beneficial uses. This complete chloroplast genome is 160,987 bp in length. In total, 130 genes were identified, including 85 protein-coding genes, 37 tRNA genes, and 8 rRNA genes. The findings of phylogenetic analysis supported that *Artocarpus* belongs to the Moraceae family and proposed a sister relationship between *Artocarpus* and *Morus.*

*Artocarpus tonkinensis* A. Chev. ex Gagnep. belongs to the genus *Artocarpus* J. R. Forst. & G. Forst. (Family: Moraceae) (Zhou and Michael 2003)). It is an evergreen tree that is up to 14–16 m high and is mainly distributed in various countries such as Cambodia, northern Vietnam, and China (Fujian, Guangdong, Guangxi, Guizhou, Hainan, and southern Yunnan) (Zhang et al. 1998; Zhou and Michael 2003). *A. tonkinensis* has very hardwood, and its fruits are edible and sweet in taste (Zhang et al. 1998; Zhou and Michael 2003). It has a variety of beneficial medicinal uses; its leaf decoction is used as a treatment for joint disorders and backache in northern Vietnam (Thuy et al. 2017; Orecchini et al. 2020). Besides, it is also an ornamental street tree with a big and dense canopy. In the present study, we assembled and characterized the complete chloroplast genome of *A. tonkinensis*. These findings will enrich the gene information and certainly contribute to the further study of this species.

The fresh leaves of *A. tonkinensis* were collected from Qinzhou City (Guangxi, China; 22° 17’46″N, 108°41′47″E). Voucher specimens were deposited at the herbarium of Guangxi Forestry Research Institute (Mr. Li, zzcx_gfri@163.com) (registration number: 2021081302), whereas the DNA samples were stored at Guangxi Key Laboratory of Special Non-wood Forest Cultivation and Utilization (Nanning, China). A sample’s total genomic DNA was extracted from about 100 mg fresh leaves by using a modified CTAB method (Doyle and Doyle 1987). Constructed the libraries with an average length of 350 bp by using the NexteraXT DNA Library Preparation Kit (Illumina, San Diego, CA), then the libraries were sequenced on Illumina Novaseq 6000 platform. Raw sequence reads were edited by using NGS QC Tool kit (Patel and Jain 2012), clean data were *de novo* assembled by SPAdes v.3.11.0 software (Bankevich et al. 2012). Finally, the assembled complete chloroplast genome was annotated via PGA (Qu et al. 2019) and submitted to GenBank (accession number: MZ379793).

The length of the complete chloroplast genome of *A. tonkinensis* was 160,987 bp, with a total GC content of 35.8%. The complete chloroplast consisted of a large single copy (LSC) region of 89,551 bp, a small single copy (SSC) region of 20,072 bp, and two inverted repeats (IR) regions of 25,682 bp. The complete chloroplast genome contained a total of 130 genes, including 85 protein-coding genes, 37 tRNA genes, and 8 rRNA genes.

Complete chloroplast genomes of 19 other species were selected to confirm the phylogenetic position of *A. tonkinensis*, with *Cinnamomum camphora* and *Magnolia liliiflora* as outgroups. All of these complete chloroplast sequences were aligned by the MAFFT version 7.429 software (Katoh and Standley 2013) and trimmed by TrimAl (Capella-Gutierrez et al. 2009). A maximum-likelihood (ML) tree was inferred by ultrafast bootstrapping with 1000 replicates through IQ-TREE 1.6.12 (Nguyen et al. 2015) based on the TVM + F+R2 nucleotide substitution model, which was selected by ModelFinder (Kalyaanamoorthy et al. 2017). The findings of the phylogenetic analysis supported that *Artocarpus* belongs to the Moraceae family and proposed a sister relationship between *Artocarpus* and *Morus* (Figure 1).

**Figure 1.**
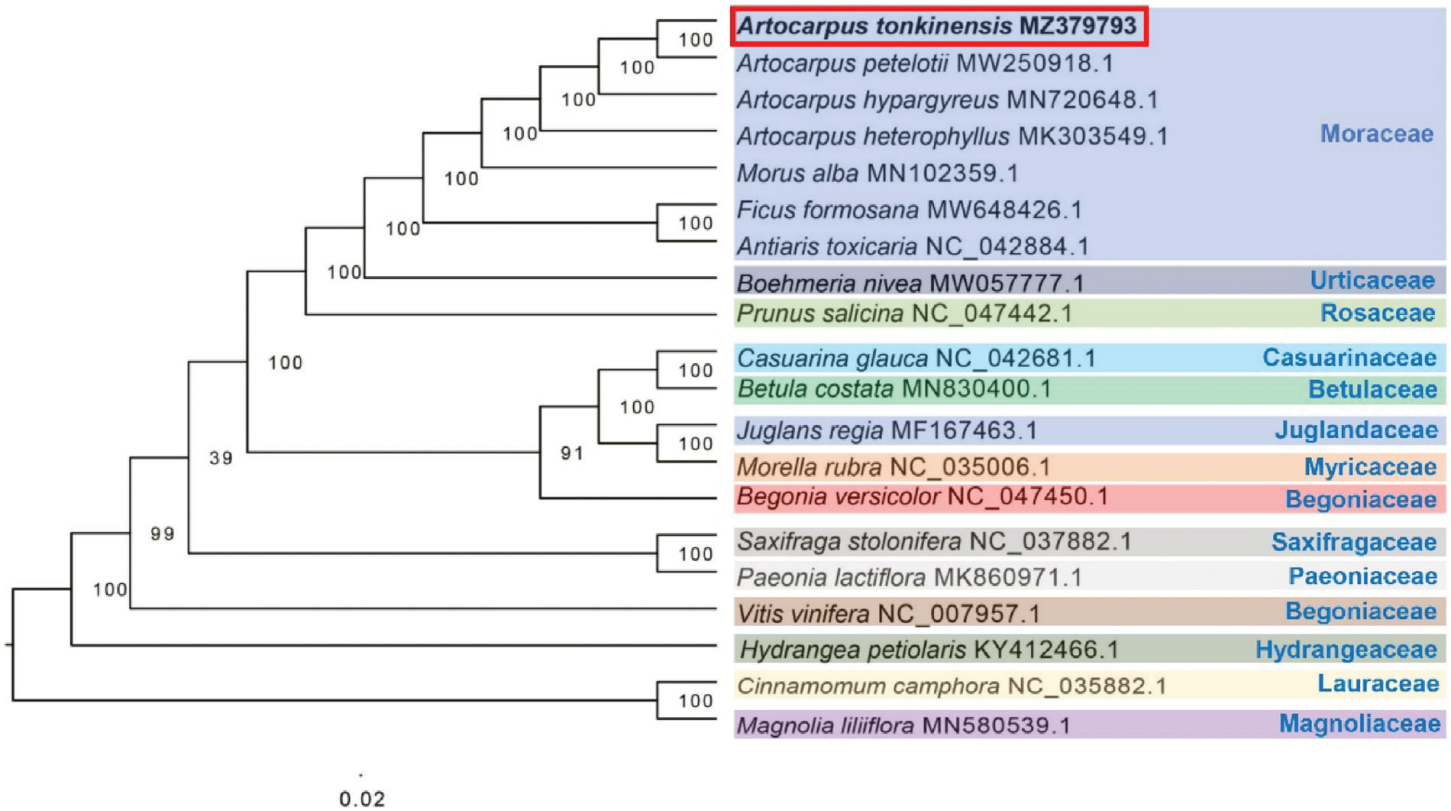
The ML phylogenetic tree based on the complete chloroplast genomes of *Artocarpus tonkinensis* and other 19 species, with *Cinnamomum camphora* and *Magnolia liliiflora* as outgroups. Numbers near the nodes represent ML bootstrap value.

## Data Availability

The data that support the findings of this study are openly available in GenBank number MZ379793 (https://www.ncbi.nlm.nih.gov/nuccore/MZ379793.1/) and SRA number PRJNA738269 (https://www.ncbi.nlm.nih.gov/Traces/study/?acc=PRJNA738269).
